# Migration routes and adult survival of the critically endangered yellow-breasted bunting *Emberiza aureola*

**DOI:** 10.1038/s41598-024-83138-4

**Published:** 2024-12-23

**Authors:** Wieland Heim, Yury Anisimov, Marc Bastardot, Batmunkh Davaasuren, Gleb Nakul, Valentina Anisimova, Nyambayar Batbayar, Ilka Beermann, Thiri Dae We Aung, Leo Damrow, Tuvshinjargal Erdenechimeg, Steffen Hahn, Arend Heim, Ramona Julia Heim, Norbert Hölzel, Friederike Kunz, Aleksey Levashkin, Martha Maria Sander, Wangworn Sankamethawee, Alexander Thomas, Johannes Kamp

**Affiliations:** 1 Institute for Biology and Environmental Sciences, University of Oldenburg, Oldenburg, Germany; 2 Department of Evolutionary Biology and Environmental Studies, University of Zürich, Zürich, Switzerland; 3 Institute of Landscape Ecology, University of Münster, Münster, Germany; 4 Ulaanbaatar, Mongolia; 5 Lausanne, Switzerland; 6 Wildlife Science and Conservation Center, Ulaanbaatar, Mongolia; 7 Institute of Biology, Syktyvkar, Komi Republic Russia; 8 Irkutsk State University, Irkutsk, Russia; 9 Biodiversity And Nature Conservation Association (BANCA), Yangon, Myanmar; 10 Swiss Ornithological Institute, Sempach, Switzerland; 11 Lab of Ornithology, Institute of Biology, University of Latvia, Riga, Latvia; 12 Kassel, Germany; 13 Nizhny Novgorod, Russia; 14 Alfred-Wegener-Institute, Potsdam, Germany; 15 Department of Environmental Science, Faculty of Science, Khon Kaen University, Khon Kaen, Thailand; 16 Werbeliner See Nature Reserve, Zwochau, Germany; 17 Department of Conservation Biology, University of Göttingen, Göttingen, Germany

**Keywords:** Conservation, East Asian flyway, Geolocator, Return rate, Ringing, Tracking, Animal migration, Conservation biology

## Abstract

Migratory animals rely on multiple sites during their annual cycles. Deteriorating conditions at any site can have population-level consequences, with long-distance migrants seen as especially susceptible to such changes. Reduced adult survival caused by persecution at non-breeding sites has been suggested a major reason for the catastrophic decline of a formerly abundant, long-distance migratory songbird, the Yellow-breasted Bunting *Emberiza aureola*. However, it is unknown whether the ongoing extinction of this Eurasian species especially in the west of its range could be related to differences in survival or migration routes. We investigated survival rates of populations from both western and eastern parts of the breeding range and successfully tracked the migration of individuals from two eastern populations with light-level geolocators. We found moderate apparent local survival rates in eastern populations, but observed no returning birds in western populations. Our tracking data highlights (1) a joint migration corridor of eastern populations through eastern China, (2) long autumn stopovers likely used for moult and re-fuelling, and (3) very long occurrences at wintering sites. These areas should be given priority for future conservation measures. We call for an increased monitoring of adult survival and breeding output in multiple populations (including western ones) of this critically endangered species to determine (1) the causes for the observed differences in apparent local survival and (2) whether the current survival rates are sufficient to sustain viable breeding populations.

## Introduction

Animal migration has evolved to optimise the use of seasonal resources^1^. Migratory species can move for more than ten thousand kilometres between breeding and non-breeding sites to exploit peaks in resource availability^2,3^. The benefits of long-distance migration can easily be lost once conditions at key sites utilised during the annual cycle are changing^4^. Such changes, e.g. in environmental conditions at important non-breeding sites, can lead to increased migration distances with population-level consequences^5,6^. Through carry-over effects, changing conditions at one location can lead to reduced survival rates at subsequent locations, and finally population declines^7–9^.

Globally, migratory bird populations have been declining more rapidly than those of resident species^10–13^. It is often unknown which factors bottleneck these populations and in which part of the annual cycle, and how these limiting factors could be mitigated^12,14^.

The Yellow-breasted Bunting *Emberiza aureola* is a formerly superabundant and widely distributed long-distance migrant that declined dramatically during the past decades and is therefore now listed as Critically Endangered^15^. The species breeds in natural wetlands, grasslands, on abandoned cropfields and along forest edges in northern Eurasia^16–18^. All populations migrate to South-East Asia during the boreal winter, where they are found in paddy fields and natural grasslands^17,18^. Between 1980 and 2013, the Yellow-breasted Bunting population declined by ca. 90%, and its breeding range retracted eastwards by 5000 km^15,19^. Population models suggested that reduced adult survival due to trapping for food in East Asia contributed significantly to the rapid population decline^19^. Data from times prior to the decline revealed relatively high apparent survival rates in Yellow-breasted Buntings compared to other small songbird species at a breeding site in Central Siberia^20^. However, recent data on survival from multiple sites across the breeding range remain unavailable.

Since 2014, the species has been reported again from areas in the western part of its range where it was believed extinct^21^. Local increases from the east of the range have been found since 2015^22^, although it remains unclear how much of this potential recovery is attributable to increased survey effort. Populations at the north-western range limit continued to decline between 2011 and 2022^23^. The strong decline of the Yellow-breasted Bunting^19^ sparked worldwide media interest and conservation actions^24^, and led to the publication of an international single species action plan for the Yellow-breasted Bunting under the Convention for Migratory Species^25^. In China, where most of the illegal trapping occurs, the species was placed in the highest category of nationally protected species in 2021, and law enforcement has been strengthened since then^24^.

Geolocator tracking of Yellow-breasted Buntings breeding in the Russian Far East has demonstrated that some individuals of this population show prolonged stop-overs during autumn migration in north-eastern China that is known as a hotspot of illegal bunting trapping^24,26^. However, it remains unknown whether all Yellow-breasted Bunting populations, including those breeding at the western edge of the range, migrate through this persecution hotspot with potentially harsh consequences for population survival rates. The longer migration route, that is coupled with reduced time in the breeding area^27^, could render western Yellow-breasted Bunting populations more susceptible to any additional pressures.

Therefore, we colour-ringed adult Yellow-breasted Buntings in five breeding populations across the breeding range to investigate survival, and equipped birds with light-level geolocators to track their migration. The aims of our study were to (1) quantify current survival rates of Yellow-breasted Buntings from populations in both the western and eastern parts of its breeding range and (2) investigate population-specific migration routes. These issues adhere to the single species action plan that proposes the development of a population model (for which information on survival is needed) and calls for a better understanding of the migratory connectivity of different Yellow-breasted Bunting populations^25^.

## Results

### Annual survival

**Fig. 1:**
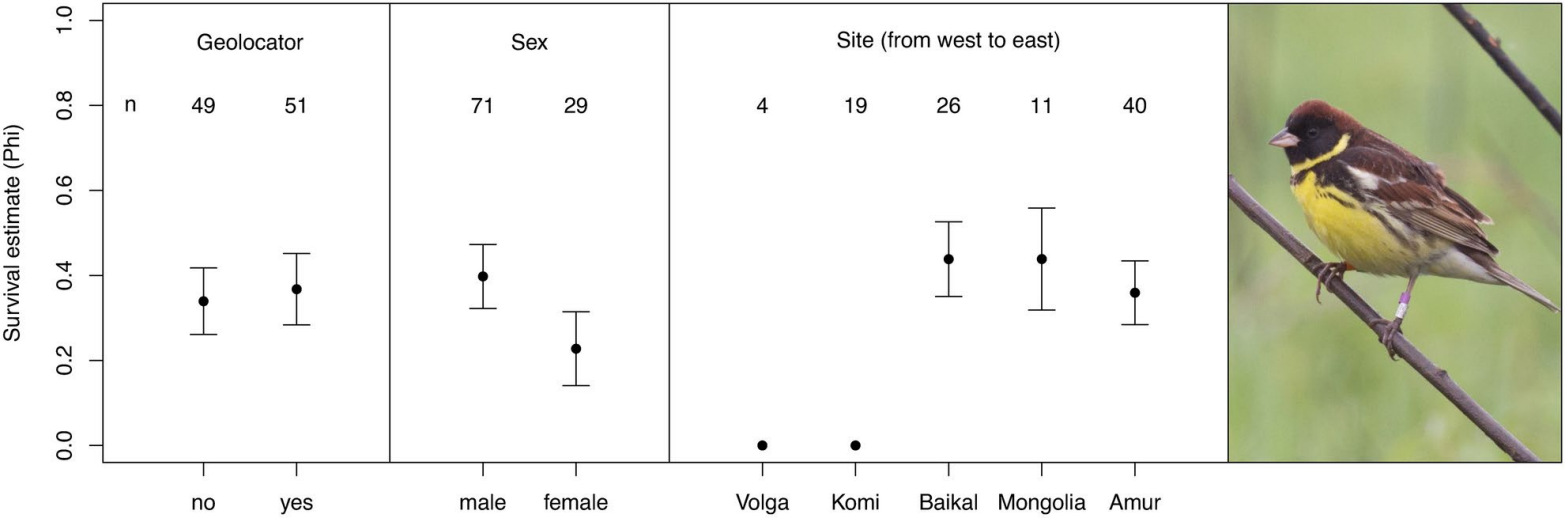
Predicted apparent annual survival estimates of colour-ringed Yellow-breasted Buntings based on models containing one of the following predictors: geolocator deployment (yes/no), sex, study site. Sample sizes for each group are given above the plotted estimates, whiskers depict standard errors. Photo of a male Yellow-breasted Bunting with individual colour-ring combination at Muraviovka Park, Amur region, Russia, May 2016, by Arend Heim.

We modelled apparent annual survival and encounter probability based on observations of colour-ringed adult Yellow-breasted Buntings (n = 100) from five different breeding populations as a function of site, sex, geolocator deployment and age (Table 1, Fig. 1). The model containing only site as predictor for apparent annual survival probability (Phi, φ) showed the highest AIC score, but was only marginally better compared to models that additionally contained the predictors sex or geolocator (Table 2). Encounter probability (P) was not affected by any of the factors in the best three models (*P* = 0.67 ± 0.14).

**Table 1:** Location, habitat, study area size, estimated breeding population (in pairs/singing males), survey years, earliest spring arrival dates, and numbers (N) of colour-ringed (CR) and geolocator-tagged (GL) Yellow-breasted Bunting at the five study sites (from west to east). Population estimate of the Amur population based on Richter et al.^28^.

Region	Volga	Komi	Baikal	Mongolia	Amur
Site name	Vetluga	Syktyvkar	Kabansky zakaznik	Khurkh bird ringing station	Muraviovka Park
Latitude	57°46 N	61°48 N	52°18 N	48°32 N	49°55 N
Longitude	45°27 E	51°55 E	106°25 E	110°32 E	127°40 E
Habitat	Abandoned fields	Floodplain meadows	Wetlands, pastures	Wetlands	Wetlands
Area (ha)	800	2488	1300	300	6500
Population	15–25	60	50	15	700
N colour-ringed	5	19	26	59	47
Years CR	2019–2020	2018–2021	2018–2021	2016–2023	2015–2018
N geolocators	3	13	17	0	18
N retrieved	0	0	6	NA	3
Years GL	2019	2019	2018–2019	NA	2016
Arrival	NA	25 May	NA	20 May	7 May

N retrieved = number of geolocators successfully retrieved > 1 year after tagging, Years GL = years in which birds were tagged with geolocators.

**Table 2:** Factors that were included in the three best Cormack-Jolly-Seber models (ΔAIC < 2) to estimate apparent survival probability (Phi, φ) and encounter probability (P) of colour-ringed Yellow-breasted Buntings, as well as corresponding AIC_c_ values of these models.

φ	P	AICc	ΔAIC
Site	1	161.57	0.00
Sex + Site	1	162.58	1.01
Geolocator + Site	1	162.69	1.12

1 = no predictor considered. See Supplement 1 for an overview of all candidate models.

Differences in apparent annual survival estimates (φ) between individuals tagged with geolocators and those without were marginal (Fig. 1), with slightly higher (but insignificant, upper [ucl] and lower confidence limits [lcl] largely overlapping) apparent survival estimates for birds with geolocators (φ = 0.37 ± 0.08, lcl = 0.22, ucl = 0.54) compared to birds without (φ = 0.34 ± 0.08, lcl = 0.21, ucl = 0.50). We found a higher apparent annual survival rate for males (φ = 0.40 ± 0.08, lcl = 0.26, ucl = 0.55) compared to females (φ = 0.23 ± 0.09, lcl = 0.10, ucl = 0.44). The most important factor explaining differences in apparent survival was the study site: not a single individual was resighted at the western study sites in the regions of Volga (φ = 0.00, lcl = 0.00, ucl = 0.00) and Komi (φ = 0.00, lcl = -0.00, ucl = 0.00), while survival estimates for the eastern study sites in the Baikal region (φ = 0.44 ± 0.09, lcl = 0.28, ucl = 0.61), Mongolia (φ = 0.44 ± 0.12, lcl = 0.23, ucl = 0.70) and Amur region (φ = 0.36 ± 0.08, lcl = 0.23, ucl = 0.52) were much higher and similar to each other (largely overlapping confidence limits, see also Fig. 1).

We found that the variable age was not included in any of the best models. A full list of all model combinations and corresponding AIC_c_ values can be found in Supplement 1.

### Migration

**Fig. 2:**
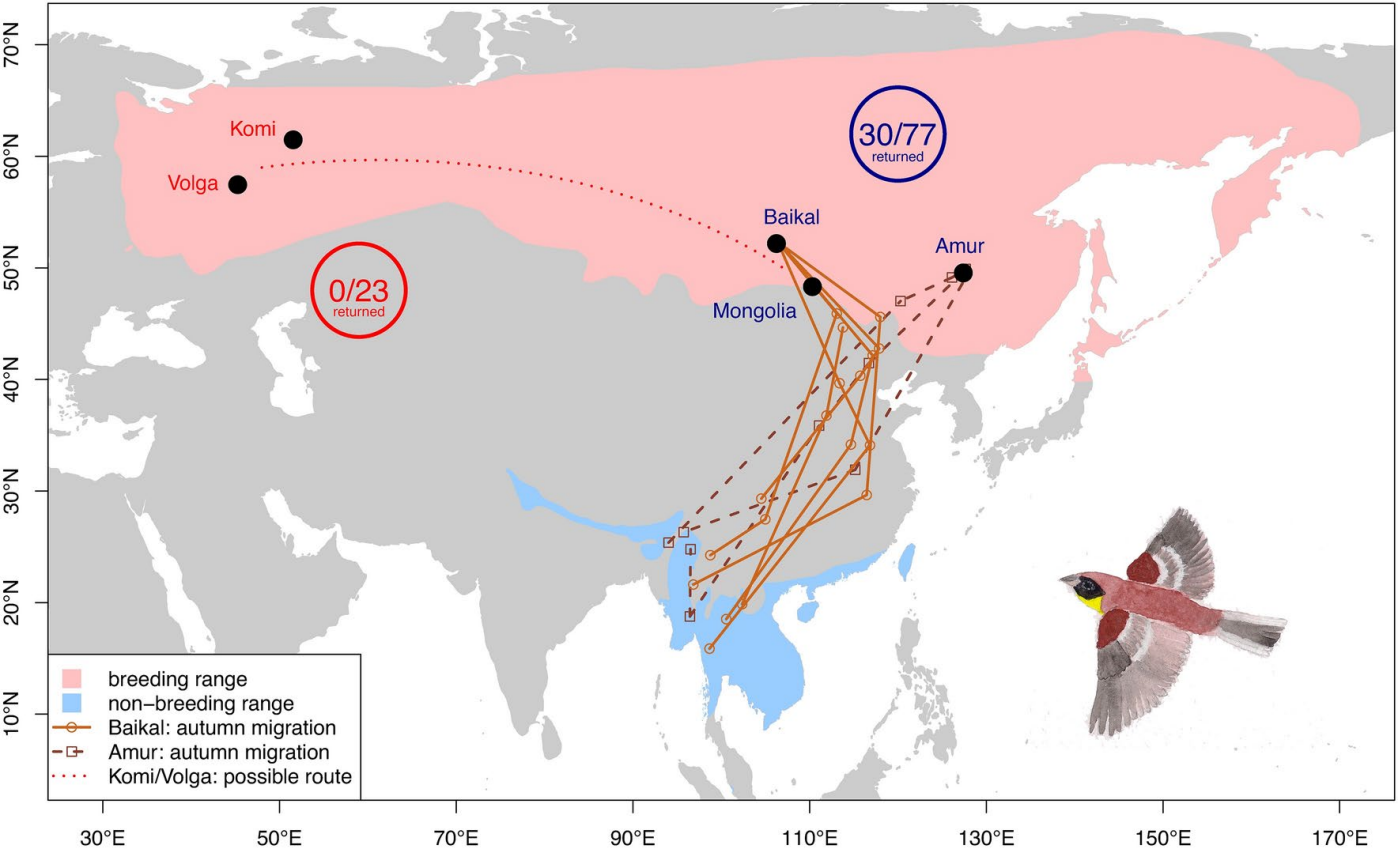
Estimated positions and possible autumn migration routes of Yellow-breasted Buntings from the Baikal and Amur breeding populations based on light-level geolocation. The total numbers of colour-ringed / relocated adults are given for the western (Komi and Volga; red) and eastern (Baikal, Mongolia and Amur; blue) populations. A great circle line was added to depict a possible migration route of the western populations that would connect with the tracked routes of the eastern populations. Breeding and wintering ranges taken from BirdLife International (2024).

We retrieved information on migration routes and timing of nine male Yellow-breasted Buntings from the breeding populations in the Amur and Baikal regions based on light-level geolocation. No data on migration could be retrieved from the western populations (Fig. 2).

**Fig. 3:**
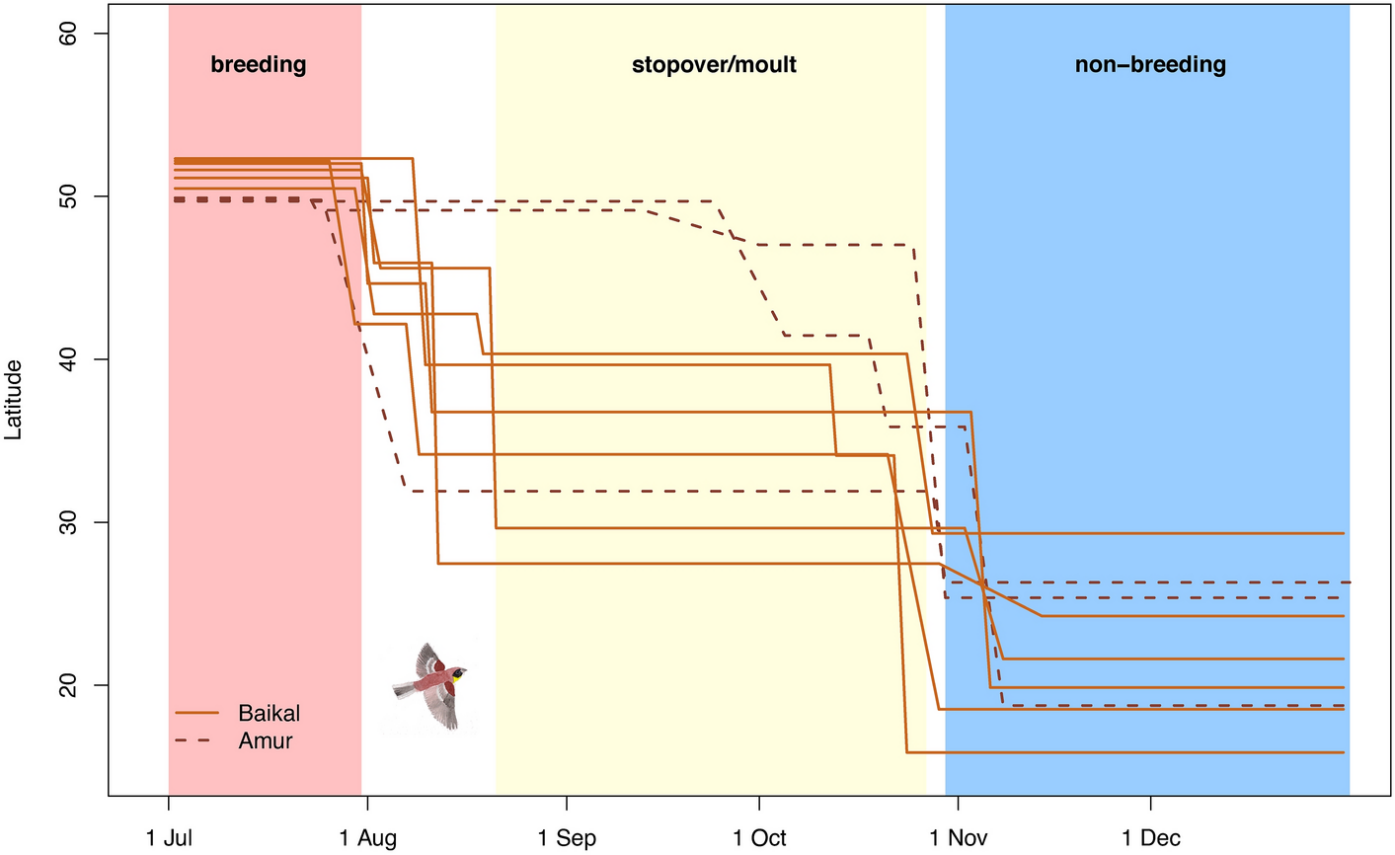
Estimated mean latitudes of stationary sites during migration of Yellow-breasted Buntings from the Baikal and Amur breeding populations based on light-level geolocation. Coloured backgrounds for seasonal phases are drawn based on median arrival and departure dates for both populations combined. Data only shown after 1 July (when most tracking devices started recording) and until 31 December (as most tracking devices have ceased to record thereafter). Each line depicts one individual.

Birds from both regions departed from the breeding sites during the last ten days of July or the first days of August (Fig. 3), except for a single individual from the Amur region that stayed close to the breeding area until 23 September (Table 3). This individual was also the only bird that did not conduct a very long autumn stopover, whereas all other individuals had a stopover between two and almost three months in north-eastern China (mean 71 days, range 51 – 84 days, n = 8). These very long stopovers occurred exclusively at sites in China between 27°N and 49°N (Fig. 3). For the birds from the Amur region, this long stopover was the first stopover, whereas birds from the Baikal population had one shorter stopover (mainly in Mongolia or northern China) before the long stopover (Table 3). Birds from both populations used two autumn stopover sites (only one individual with only one stopover) before reaching their wintering sites in late October or early November (median 29 October, range 24 October–14 November, n = 9). We observed no differences in the total duration and the total distance of autumn migration between the two populations (Table 3). Stopover time was also similar between the two populations, but travel time was shorter and, consequently, travel speed higher for birds from the Baikal population (Table 3).

**Table 3:** Migration parameters of male Yellow-breasted Buntings from two breeding populations in the Baikal and Amur regions, Russia.

Population	Baikal	Amur
**Autumn migration**		
Post-breeding departure	31 Jul (26 Jul–08 Aug, n = 6)	24 Jul (22 Jul–23 Sep, n = 3)
Arrival first stopover	02 Aug (30 Jul–10 Aug, n = 6)	06 Aug (23 Jul–04 Oct, n = 3)
Duration first stopover (d)	13 (8–63, n = 6)	51 (13–82, n = 3)
Departure first stopover	14 Aug (07 Aug–12 Oct, n = 6)	17 Oct (12 Sep–27 Oct, n = 3)
Arrival second stopover	15 Aug (09 Aug–13 Oct, n = 6)	10 Oct (30 Sep–20 Oct, n = 2)
Duration second stopover (d)	73 (9–84, n = 6)	18 (12–24, n = 2)
Departure second stopover	26 Oct (21 Oct–03 Nov, n = 6)	28 Oct (24 Oct–01 Nov, n = 2)
Minimum number of stopovers	2 (n = 6)	2 (1–2, n = 3)
Stopover time (d)	85 (72–93, n = 6)	75 (25–82, n = 3)
Travel time (d)	9 (5–18, n = 6)	20 (15–24, n = 3)
Total duration (d)	97 (77–105, n = 6)	97 (45–99, n = 3)
Total distance (km)	4184 (3105–4982, n = 6)	4219 (3990–4419, n = 3)
Migration speed (km/d)	45 (35–64, n = 6)	61 (40–98, n = 3)
Travel speed (km/d)	531 (202–984, n = 6)	223 (166–283, n = 3)
**Wintering**		
Arrival wintering	02 Nov (24 Oct–14 Nov, n = 6)	29 Oct (29 Oct–07 Nov, n = 3)
Duration wintering (d)	178 (159–182, n = 3)	145 (n = 1)
Number of sites	1 (n = 6)	1 (1–2, n = 3)
**Spring migration**		
Departure wintering	23 Apr (15 Apr- 24 Apr, n = 3)	01 Apr (n = 1)
Arrival first stopover	27 Apr (25 Apr–30 Apr, n = 2)	NA
Duration first stopover (d)	3 (2–3, n = 2)	NA
Departure first stopover	30 Apr (28 Apr–02 May, n = 2)	NA
Arrival last stopover	20 May (n = 1)	NA
Duration last stopover (d)	6	NA
Departure last stopover	26 May (n = 1)	NA
Number of stopovers	4 (n = 1)	NA
Stopover time (d)	26 (n = 1)	NA
Travel time (d)	8 (n = 1)	NA
Total duration (d)	34 (n = 1)	NA
Total distance (km)	5654 (n = 1)	NA
Migration speed (km/d)	166 (n = 1)	NA
Travel speed (km/d)	707 (n = 1)	NA
Arrival breeding	28 May (n = 1)	21 May (18 May–24 May, n = 2)
Duration breeding (d)	72 (n = 1)	95 (67–122, n = 2)

Given are the median (for dates) or mean (for distances and speeds), range, and the sample size for all parameters. All information is based on geolocator tracking except for the arrival dates at the breeding grounds at the Amur site, which are based on observations of colour-ringed individuals. For details how the variables were calculated please refer to the methods section. d = days, km = kilometres.

Birds stayed at their wintering sites for up to six months throughout the northern winter (Table 3), except for one bird from the Amur population that changed its site within Myanmar between January and February. Most geolocators stopped recording at the end of the wintering season or during early spring.

One bird from the Amur population spent 145 days in the wintering area, whereas three birds from the Baikal population stayed 159 to 182 days (Table 3). This was caused by differences in the onset of departure from wintering sites: the individual from the Amur population left by 1st of April, whereas the birds from the Baikal population departed in the second half of April (Table 3).

Complete spring migration data were obtained for only one individual from the Baikal population. This bird used four different stopover sites and reached the breeding grounds at the 28th of May (Supplement 4). Two of the males from the Amur population where already observed at their breeding sites at the 18th and 24th of May, respectively. We estimated that Yellow-breasted Bunting males stayed for 67 (Amur) and 72 (Baikal) days at their breeding sites, and a single bird from the Amur site stayed for 122 days (Table 3). Individual maps of all tracked individuals can be found in Supplement 4.

### Overlap with persecution sites

**Fig. 4:**
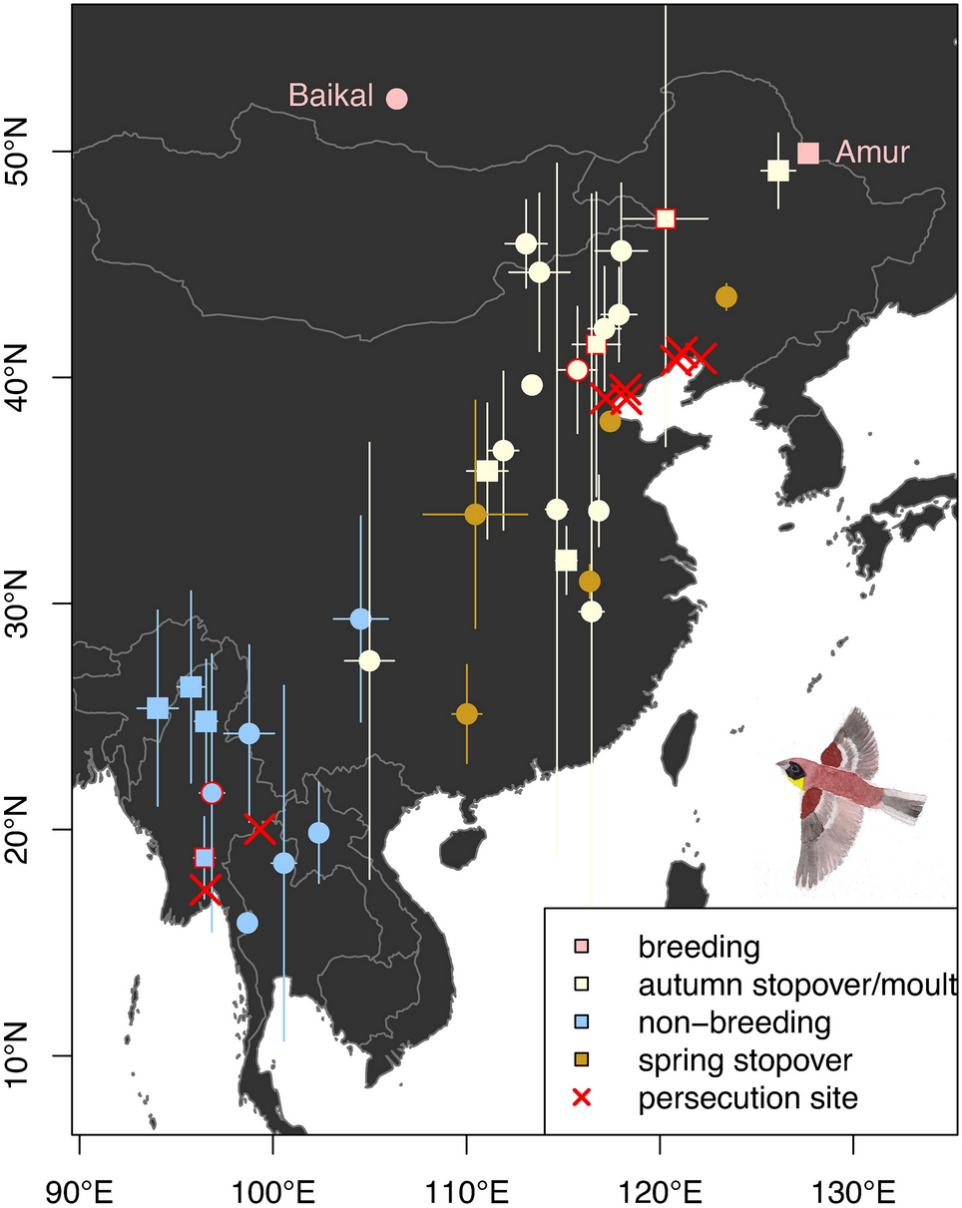
Estimated stationary positions of Yellow-breasted Buntings from the Baikal (points) and Amur (squares) breeding populations and location of sites with known persecution (trapping or hunting events, based on Heim et al. 2021, Kamp et al. 2015, supplemented). Whiskers indicate the standard deviation around the mean latitudes and longitudes. Highlighted with a red border are those stationary sites (calculated as rectangles centred around the mean estimated position plus the standard deviation of these coordinates) that overlapped with known sites of persecution.

We compiled nine cases of persecution of Yellow-breasted Buntings (Supplement 3). Six of these hunting sites were situated in eastern China around the Bohai bay and referred to the autumn migration period (August to September), while three cases from sites in Myanmar and Thailand referred to the wintering season (December to January). Two out of five (40%) autumn stopover positions of individuals from the Amur population overlapped with known persecution sites, and one out of 11 (9%) autumn positions of birds from the Baikal population (Fig. 4). Regarding positions during the wintering season, one out of four (25%) positions from the Amur population and one out of six (17%) positions from the Baikal population overlapped with known sites of persecution (Fig. 4). We found no evidence for persecution during spring migration, and no spring stopover positions overlapped with known hunting sites (Fig. 4).

## Discussion

### Annual survival

We found a marked difference in the number of returning birds between western and eastern Yellow-breasted Bunting populations. While the three eastern populations showed moderate survival rates (41%, n = 77 adults), not a single returning individual out of 23 marked adult birds was observed in the two western populations. While we cannot fully exclude that the lack of observations of returning individuals in the Volga population is linked to the low sample size (n = 4 adults), our multi-year effort at the Komi site (n = 19 adults) provides evidence that at least in this population the return rate must be very low.

However, it is possible that the level of site fidelity, and consequently, the apparent annual survival rate, is generally lower in the western populations, potentially linked to the human-shaped habitats they inhabit. These habitats, such as abandoned cropland and hay meadows, are most likely much less stable than the natural wetlands where most of the eastern populations breed. Yellow-breasted Buntings of the western populations might have adapted to annually changing, and thus unpredictable, habitat availability by a lower level of site fidelity^29^. Alternatively, the habitats at our two western study sites might be of lesser quality for Yellow-breasted Buntings, which could also explain differences in return rates^30,31^. On top of that, the lower population densities observed in the west of the range might also explain the lack of returning birds, as lower abundances were found to be linked to higher breeding dispersal in other bird species^32^. Interestingly, a sharp decrease in survival was also observed in a Yellow-breasted Bunting breeding population in Central Siberia before the species went extinct^20.^ While the adult annual survival rate in this region was moderately high (> 0.5) between 1989 and 1998, it strongly declined between 1998 and 2001 and was zero in 2001 and 2004, with the species becoming extinct in 2005 (Oleg Bourski, unpublished data). The lack of returning adults to our western study sites might therefore be a warning that these populations could face imminent extinction. Such a link between decreasing survival rate and subsequent population decline was also established for the closely related Reed Bunting *Emberiza schoeniclus* wintering in Great Britain^33^ and other songbird species^34^.

Contrary to our expectations and data from literature^35^, one of the best models suggested slightly higher apparent survival of birds tagged with geolocators compared to those without. Given the negligible difference we consider this as an artefact. We also found evidence for a somewhat lower apparent survival rate of female versus male Yellow-breasted Buntings. This is a common pattern in birds and has been linked to sexual differences in the costs of breeding^36^. On top of that, the potentially lower survival of females might be explained by a general lower level of philopatry in female versus male birds^37^.

While the survival rate of adult Yellow-breasted Buntings of the eastern populations seems to be much higher compared to western populations, it is still lower than survival rates estimated for this species before the decline (0.54, Bourski et al. 2015) and much lower compared to the ecologically similar (i.e. long-distance migrant) Ortolan Bunting *Emberiza hortulana*, for which survival rates of > 0.62 were reported^38,39^. An increased monitoring of adult survival and breeding output in multiple populations (including western ones) of this critically endangered species would be desirable to determine 1) the causes for the observed differences in apparent survival and 2) whether the current survival rates are sufficient to sustain viable breeding populations.

### Migration

We found that Yellow-breasted Buntings from eastern breeding populations in the Baikal and Amur regions were following a similar migration corridor through eastern China towards wintering sites in South-East Asia and southern China (Fig. 2).

Most interesting are the very long (more than two months) stopovers of almost all individuals of both populations in China during autumn migration. No such long stopovers were detected during spring migration, although the location of the spring stopover sites was similar to the sites used during autumn migration (Fig. 4). The one individual (21TE) that was tracked for a full annual cycle showed a slightly more eastern route during spring compared to autumn (Supplement 4). The timing (late August to late October) of the long autumn stopovers coincides with the post-nuptial moult in this species^40–42^. We therefore consider it most probably that most (though not all) adult Yellow-breasted Buntings leave their breeding sites before moult commences, and undergo a complete moult at stopover sites in north-eastern China. The occurrence of moult at stopover sites in eastern China was confirmed by skins of actively moulting adults^42,43^. Further evidence is provided by observations further (south-)east, at a study site in South Korea, where exclusively freshly moulted adults were observed during autumn migration^44^ and at the wintering grounds in Thailand, where adults arrive with newly moulted primary feathers (WS, unpublished data). It has been assumed that only western Yellow-breasted Buntings moult at stopover sites (intra-migratory moult), while birds from eastern populations with the shortest migration routes may moult at their breeding sites (pre-migratory moult,^42^). However, our data suggests that none of the birds from the Baikal region and only one out of three males from the Amur region has moulted in the breeding area. Moult at stopover sites has recently been found for other songbirds migrating along the East Asian flyway^45,46^, likely linked to temporal constraints in resource availability at their highly seasonal Siberian breeding sites. Future studies should investigate whether the individual decision to moult at breeding or stopover sites might be linked to breeding success, as has been observed in other bird species^47,48^.

For many individuals, the time spent at these moulting sites is longer compared to the time spent at their breeding sites. During this time, they most likely not only moult, but may also exploit good conditions at the stopover site for accumulating sufficient fuel stores for their final migratory flight. Alternatively, these long stopovers might also serve to monitor the conditions at the wintering grounds from afar (e.g. the end of the rainy season and start of rice harvest in South-East Asia) in order to time their arrival aligned to resource availability, as has been suggested for other small songbirds occurring in seasonal habitats^49,50^. Bird trappers likely take advantage of (1) the long stationary phases with restricted movement due to flight feather moult (increasing the availability and predictability for efficient hunting) and (2) the following physiological stage of migratory fat accumulation (which benefits the artificial fattening before they are sold as food, Heim et al. 2021) during this part of the Yellow-breasted Bunting´s annual cycle. This highlights the conservation importance of these stopover areas, and future studies using GPS devices would be needed to pin-point precise locations.

The wintering locations of Yellow-breasted Buntings from the Baikal and Amur populations overlapped and covered a considerable proportion of the species´ known and predicted seasonal range in South-East Asia and southern China^15,26^. However, our tracked individuals migrated neither to the westernmost parts of the wintering range in Nepal or India nor to the easternmost parts of the range between Hainan Island and Taiwan. Given the patterns of migratory connectivity observed in other Asian landbirds^26^, it is possible that Yellow-breasted Buntings from the west of the breeding range spend the winter in the western part of the range, and that birds from the eastern breeding populations, e.g. from Japan or Kamchatka peninsula, migrate to wintering sites in the eastern part of the range. Future tracking studies should investigate the routes of these peripheral populations. From a conservation perspective, the wintering sites warrant special attention, as no other sites during the annual cycle were used for such a long time by the tracked Yellow-breasted Buntings in our study, with individuals from the Baikal population staying up to six months. A mid-winter movement from southern Myanmar to northern Myanmar was detected in only one individual from the Amur population. Such northward movements during late winter were also documented for ringed birds in Thailand, and were interpreted as preparatory movements before the start of the long-distance flights to their breeding grounds^51^.

At the breeding sites, we found that birds from the Amur and Baikal regions stayed for only two to four months. This is still much longer compared to birds from a breeding population further north-west in Central Siberia (58 days,^20^). Birds from the westernmost populations likely have even less time at their breeding sites due to later spring arrival dates (Table 1), which could limit their possibilities for replacement or second clutches, which are only known from eastern Yellow-breasted Buntings^27^. Later arrival in spring has been linked to lower reproductive output in many bird species^52–55^. If productivity would indeed be lower, western Yellow-breasted Buntings might be much more susceptible to any further pressures such as the reduction of adult survival, which could explain why declines and local extinctions are much more pronounced in the west of the species´ breeding range.

The higher autumn travel speeds of Yellow-breasted Buntings from the Baikal compared to the Amur population might be an adaption to compensate for the later departure from the breeding site, caused by a later onset of spring (and, consequently, later breeding,^27^) further west and north due to a more continental climate.

No tracking data was obtained from individuals of the western breeding population, but a westward route that connects with the migration corridor of the eastern populations through eastern China seems plausible, as ring recoveries of Yellow-breasted Buntings from distant breeding locations in Finland in the west and Yakutia in the east at wintering sites in Thailand suggest a joint wintering area^26,51^. Other songbirds breeding in western Siberia, such as Arctic Warblers *Phylloscopus borealis,* are also known to migrate longitudinally towards the Lake Baikal area before turning southward through Mongolia to reach their wintering sites in South-East Asia^46^.

### Persecution and other possible threats

We showed that tracked individuals from both the Baikal and Amur populations did indeed visit areas where persecution of the species occurs (Fig. 4). We also demonstrated that persecution is not only imminent at autumn stopover sites in China^24^, but also prevalent at wintering sites of both eastern populations. and possibly much more widespread than our limited data suggest e.g. in Myanmar^56^, Nepal^57^, Thailand (WS, pers. obs.) and potentially in eastern Uttar Pradesh, Bihar and parts of Jharkhand, India as well as Laos^25^. Consequently, given the wide occurrence of persecution during the non-breeding season, we consider the level of persecution to be similar among Yellow-breasted Bunting populations. A stricter legalisation and law enforcement to stop the persecution of Yellow-breasted Buntings is therefore needed throughout its non-breeding range, which will likely benefit all breeding populations and many other species^58,59^.

The lack of evidence for persecution during spring migration might hint at reduced persecution pressure during this season, although it needs to be highlighted that spring stopover sites were only available from two individuals in our study. Nevertheless, lower spring persecution pressure could be explained by faster spring migration (shorter total migration duration) and shorter stopover duration as observed in one of our tracked individuals (Table 3), which could render the occurrence of the species less predictable for bird trappers.

The population development of the Yellow-breasted Bunting is unlikely to be dependent on current persecution levels alone. While changes in the availability of breeding habitat are considered unlikely^19,23^, changes in the conditions at its south-east Asian wintering sites might affect population densities as well^20^, and might technically be inseparable from losses due to persecution^19^. Large aggregations of several thousands of Yellow-breasted Buntings have recently been observed at roosting sites in Cambodia, Myanmar and Thailand during the wintering season^56,60,61^, where the species could suffer from habitat loss due to (1) conversion of natural grasslands to agricultural areas^56^, (2) grassland fires^56,60^ and (3) reed harvest^56^. Direct and indirect effects of increased pesticide use could be another limiting factor for Yellow-breasted Buntings feeding in agricultural areas, as has been observed in other bunting species in Europe^62–64^ and North America^65,66^. Future studies should investigate the importance of these additional threats.

### Conclusions

We found significant differences in the number of returning birds between western and eastern Yellow-breasted Bunting populations that link well with the range contraction and local extinctions in the west of the species´ breeding range. We demonstrate that birds from two eastern populations use a joint migration corridor through eastern China but found no evidence for population-specific migration routes or wintering sites. Consequently, we consider the level of persecution to be similar between populations. We suggest that additional life-history constraints caused by a longer migration route, such as less time at the breeding sites and a higher probability to encounter adverse conditions during migration, could lead to the stronger population declines in the west of the range. Site-based conservation should focus on moulting and wintering sites where the birds spent most of the time during their annual cycle. Banning the persecution of Yellow-breasted Buntings in all range countries still must be considered as a key action to increase adult survival to magnitudes that would sustain breeding populations. On top of that, future research should investigate further potential limiting factors for the population of this critically endangered species, such as habitat loss or pollution on the wintering grounds.

## Methods

### Fieldwork

To investigate survival, we performed a colour-ring study at five Yellow-breasted Bunting breeding sites in Russia and Mongolia spread between the westernmost populations in European Russia and populations from the Russian Far East. At the study sites in the Amur, Baikal, Komi and Volga regions (Fig. 2), we trapped birds in their territories during the breeding season (May–July) with mist-nets, attracting them with playback of the species’ song between 2015 and 2020. We also used a self-made decoy of a male Yellow-breasted Bunting to attract birds to the nets. All territories where individuals were colour-ringed were visited again in one or several subsequent years (2016–2023) to search for returning birds, and playback was used again. Search efforts for colour-ringed birds at all study sites were targeted at suitable habitats in a radius of at least one kilometre around the ringing sites. At the study site in Mongolia, we trapped birds in standard nets of the Khurkh bird ringing station between 2016 and 2023. At all sites, we marked birds with one numbered metal ring and an individual combination of three colour rings (i.e. two rings per leg) which enabled identification in the field. Birds were aged as juvenile (i.e. birds hatched in the year of the study), second-year birds (i.e. hatched in the year before the year of study) or older birds (hatched earlier as the year before the year of study) based on Svensson (1992). For an overview of the location, habitat, and fieldwork activities in the five study areas see Table 1. The summed capture effort at each site was linked to the size of the local breeding population. For example, more time was spent for fieldwork in the large breeding population at the Amur site compared to the very small population at the Volga site. And while we were able to check all local males for colour-rings in the western populations, this was not possible in the Amur population. However, the effort per individual territory (of a colour-ringed bird) was similar throughout all study sites and years. Differences in local habitat use by the birds might have affected the efficiency our survey, e.g. birds breeding in low shrubs (e.g. Amur site) can be easier to observe and catch compared to birds breeding in meadows with adjacent trees (e.g. Komi site). Further details regarding the habitat use of Yellow-breasted Buntings at each of the study sites can be found in Beermann et al. (2021).

To study migration routes, we equipped 51 Yellow-breasted Buntings (49 males and 2 females) at four breeding sites with light-level geolocators (for details see Supplement 2). We used the models Intigeo P55B1-7 (Migrate Technologies, Cambridge/UK) at the Amur, Komi and Volga study sites and Intigeo P55B1-7 as well as GDL2.0 (Swiss Ornithological Institute, Sempach/Switzerland) at the Baikal site. Intigeo devices were programmed to collect data immediately after deployment, whereas GDL2.0 devices started data collection from 10 July onwards. Geolocators were mounted with a leg-loop harness made of plastic tubes with a diameter of 1 mm (50% of the individuals at the Amur site) or 1 mm wide braded nylon strings (all remaining Intigeo geolocators) or elastic rubber strings (GDL2.0 geolocators only) and had a total weight of ca. 0.7 g, which corresponded to less than 4% (3.0–3.9%) of the individual body weight (mean = 21.2 g, range 18.1–23.5 g, n = 47). Note that data from three individuals of the Amur population has been published earlier^24,26^.

All methods were carried out in accordance with relevant guidelines and regulations and are reported in accordance with the ARRIVE guidelines. Protocols for the handling, ringing and geolocator tracking of Yellow-breasted Buntings were approved by the Russian Academy of Science and adhered to the legislation of the Russian Federation, and all necessary permits were received.

### Annual survival analysis

To estimate survival of Yellow-breasted Buntings we built Cormack-Jolly-Seber-Models (CJS) in program MARK, interfaced via the R package RMark^68,69^. We assumed that individual return rates are a product of individual survival and encounter probability (P). As we had no data to correct for emigration and mortality, the term ‘apparent survival’ (Phi, φ) is used henceforth. We used unique encounter histories of colour-ringed individuals to model both P and φ. We included only individuals ringed during the breeding season between May and July, as birds captured between August and October are likely passage migrants and do not necessarily belong to the local breeding populations. We also excluded birds colour-ringed as juveniles due to low sample size (n = 3) and the fact that none of these were observed later on. The encounter histories included the identifier of the individuals and whether a bird was encountered in the years 2015–2023 (0 = not encountered in a given year, 1 = encountered, e.g., ‘0110000000’ = individual ringed in 2016, observed in 2017 but not afterwards; the first “1” always indicates the year of ringing). We included the following predictors, that could potentially affect φ and P: We expected that *age* could affect φ, since first-year Yellow-breasted Buntings might have a lower chance of breeding successfully in their first spring^20,70^ and might therefore exhibit lower levels of philopatry^71^, or they might have higher mortality rates^72^, all of which would translate into a lower φ for second-year birds compared to older birds. We also expected that whether a bird was equipped with a *geolocator* or not could affect φ, because lower apparent survival rates of tagged birds have been reported from other species^35^, and we also expected that it could affect P, as we cannot rule out that our search efforts in the year after tagging might have been biased towards geolocator-tagged birds (in order to retrieve the tags), for which a higher P could be possible for those individuals. Furthermore, we expected that φ might be affected by *sex*, as differences in apparent survival between males and females are known for many bird species^36^. Given the behavioural differences of males (singing from exposed perches) and females (hiding at the nest) during the breeding season^27^, we also expected a higher P for males. We included *site* as factor determining both φ and P, as minor differences between either observers (e.g. experience with reading colour rings), natural conditions (e.g. higher visibility of colour-ringed males in lower shrubs compared to birds singing in trees) or local abundance^32^ at the different sites could affect both probabilities. We built models with all possible combinations of predictors and compared models based on AICc values by calculating ΔAIC for all models in comparison with the model with the lowest AICc^73^. Models that had a ΔAIC of less than 2 were assumed to receive similar support from the data, and therefore have similar model fit and explanatory power given their complexity^73^.

### Migration analysis

Geolocation data was analysed largely following the light-level geolocator user´s guide^74^. Upon recapture, the battery of the Intigeo loggers had expired in all cases, whereas data for a full year were collected by one GDL2.0 geolocator. Data of the Intigeo devices were analysed with GeoLight^75^. A breeding site calibration was used to determine the sun elevation angle. Stationary sites and the migration schedule were determined with the *changeLight* function (quantile = 0.95–0.99, days = 1–3). Around two weeks around the equinox were excluded from the calculation of the coordinates with the *coord* function. The *mergeSites* function was used to merge sites that were closer to each other than the distance threshold of 200 km. Light data of the GDL2.0 geolocator was analysed with SGAT^76^. We used a breeding site calibration to estimate the sun elevation angle. We used the standards for the gamma distribution of flight speeds (beta = c(2.2, 0.08)) in the movement model and used an interpolation (tol = 0.14) to deal with noise around the equinox periods^74^. We used the *groupedTresholdModel* and 4000 iterations. Note that part of the tracking data (Amur population) was published earlier^24,26^.

We used the *distHaversine* function in R package geosphere^77^ to calculate haversine distances between coordinates. Stopover time was calculated as the total number of days spent at stopover sites during autumn and spring migration, respectively. Total duration was defined as the number of days between the departure from the breeding grounds and arrival at the wintering site for autumn migration and vice versa for spring migration. We calculated travel time by subtracting the stopover times from the total duration for autumn and spring migration, respectively. The duration of the wintering season was calculated as the number of days between arrival at the first wintering site and departure from the last wintering site. We estimated the duration of stay at the breeding site by calculating the number of days between the julian date of the departure from the breeding site in year 1 and the julian date of the arrival at the breeding site in year 2. We calculated the total distance for autumn migration by summing up the distances between consecutive sites from the breeding site to the wintering destination and vice versa for spring migration. Migration speed was calculated by dividing the total distance by the total duration for autumn and spring migration, respectively, whereas travel speed was calculated by dividing the total distance by the travel time.

### Persecution

We compiled evidence of persecution (i.e. hunting/trapping for consumption, trade, agricultural pest control or merit release) of Yellow-breasted Buntings from the recent literature^19,24,25^ and by asking local conservationists from the species´ range countries during a webinar dedicated to the monitoring of Asian landbirds held in March 2024. We included only such cases where the origin of the birds was known, not including cases of Yellow-breasted Buntings found in markets or restaurants which were unlikely to be sourced locally. Only recent cases (since 2013) were considered. We tested whether the estimated positions of geolocator-tracked individuals were overlapping with trapping sites. To account for the location error of geolocation data, we built rectangles for each estimated position centred around the mean estimated coordinates, with the width and breadth of the rectangles defined by the standard deviation of the estimated coordinates.

## Supplementary Information

Supplementary Information.

## Data Availability

All tracking data is publicly available at Movebank (https://www.movebank.org/) in the studies “Yellow-breasted Bunting (Emberiza aureola) Amur” (Movebank study ID 3536641453) and “Yellow-breasted Bunting (Emberiza aureola) Baikal” (Movebank study ID 3536688940). Data regarding survival and R scripts are available at Zenodo (10.5281/zenodo.14501764).
