# Supplementary material for: Migration routes and adult survival of the critically endangered yellow-breasted bunting Emberiza aureola
Source: Sci Rep. 2024 Dec 23;14:30593. doi: 10.1038/s41598-024-83138-4 (PMC11666740; doi:10.1038/s41598-024-83138-4)
Supplement: Supplementary file 1 — Supplementary Information. [file 41598_2024_83138_MOESM1_ESM.pdf]

Supplement to:

Migration routes and adult survival of the Critically Endangered Yellow-breasted Bunting *Emberiza aureola*

Wieland Heim[1,2,3*], Yury Anisimov[4], Marc Bastardot[5], Batmunkh Davaasuren[6], Gleb Nakul[7], Valentina Anisimova[8], Nyambayar Batbayar[6], Ilka Beermann[3], Thiri Dae We Aung[9], Leo Damrow[3], Tuvshinjargal Erdenechimeg[6], Steffen Hahn[10,11], Arend Heim[12], Ramona Julia Heim[2,3], Norbert Hölzel[3], Friederike Kunz[3], Aleksey Levashkin[13], Martha Maria Sander[14], Wangworn Sankamethawee[15], Alexander Thomas[16], Johannes Kamp[3,17]

[1]Institute for Biology and Environmental Sciences, University of Oldenburg, Oldenburg, Germany

[2]Department of Evolutionary Biology and Environmental Studies, University of Zürich, Zürich, Switzerland

[3]Institute of Landscape Ecology, University of Münster, Münster, Germany

[4]Independent researcher, Mongolia

[5]Lausanne, Switzerland

[6]Wildlife Science and Conservation Center, Ulaanbaatar, Mongolia

[7]Institute of Biology, Syktyvkar, Komi Republic, Russia

[8]Irkutsk State University, Irkutsk, Russia

[9]Biodiversity And Nature Conservation Association (BANCA), Yangon, Myanmar

[10]Swiss Ornithological Institute, Sempach, Switzerland

[11]Lab of Ornithology, Institute of Biology, University of Latvia, Riga, Latvia

[12]Kassel, Germany

[13]Nizhny Novgorod, Russia

[14]Alfred-Wegener-Institute, Potsdam, Germany

[15]Department of Environmental Science, Faculty of Science, Khon Kaen University, Thailand
[16]Werbeliner See nature reserve, Zwochau, Germany
[17]Department of Conservation Biology, University of Göttingen, Göttingen, Germany

*corresponding author: wieland.heim@uni-oldenburg.de

ORCiD:
Wieland Heim: https://orcid.org/0000-0002-3262-2491
Batmunkh Davaasuren: https://orcid.org/0000-0002-8496-1508
Gleb Nakul: https://orcid.org/0000-0001-9643-5663
Valentina Anisimova: https://orcid.org/0000-0003-3157-3111
Nyambayar Batbayar: https://orcid.org/0000-0002-9138-9626
Thiri Dae We Aung: https://orcid.org/0009-0007-8012-0481
Steffen Hahn: https://orcid.org/0000-0002-4924-495X
Ramona Julia Heim: https://orcid.org/0000-0002-2503-7075
Norbert Hölzel: https://orcid.org/0000-0002-6367-3400
Martha Maria Sander: https://orcid.org/0000-0002-9036-0450
Johannes Kamp: https://orcid.org/0000-0002-8313-6979

Supplement 1: Candidate Cormack-Jolly-Seber models fitted to estimate apparent survival (Phi) and encounter probabilities (p) of colour-ringed Yellow-breasted Buntings and corresponding AICc values. 1 = no predictor considered.

**Phi**	**P**	**AICc**	**ΔAIC**
Site	1	161.57	0.00
Sex + Site	1	162.58	1.01
Logger + Site	1	162.69	1.12
Logger + Site	Logger	164.52	2.95
Logger + Sex + Site	1	164.90	3.33
Age + Logger + Site	Logger	165.45	3.88
Sex + Site	Sex	166.34	4.77
Logger + Sex + Site	Logger	166.88	5.31
Age + Logger + Sex + Site	Logger	166.94	5.37
Age + Sex + Site	Sex	166.99	5.42
Site	Site	168.17	6.60
Logger + Sex + Site	Sex	168.73	7.16
1	1	169.04	7.47
Logger + Site	Site	169.22	7.65
Sex + Site	Site	169.38	7.81
Age + Logger + Sex + Site	Sex	169.40	7.83
Age + Sex + Site	Site	169.98	8.41
Logger + Sex + Site	Logger + Sex	170.35	8.78
Age + Logger + Sex + Site	Logger + Sex	170.37	8.80
Age + Logger + Site	Site	170.58	9.01
Sex	1	170.61	9.04
Logger	1	171.05	9.48

Age	1	171.09	9.52
Logger + Site	Logger + Site	171.59	10.02
Logger + Sex + Site	Site	171.82	10.25
Sex + Site	Sex + Site	172.21	10.64
Logger + Sex	1	172.34	10.77
Age + Logger + Sex + Site	Site	172.50	10.93
Logger	Logger	172.75	11.18
Age + Logger	1	173.15	11.58
Logger + Sex	Logger	173.70	12.13
Logger + Sex + Site	Logger + Site	174.06	12.49
Sex	Sex	174.11	12.54
Logger + Sex + Site	Sex + Site	174.69	13.12
Age + Logger + Sex + Site	Logger + Site	174.76	13.19
Age + Logger + Sex + Site	Site + Sex	175.61	14.04
Logger + Sex	Sex	176.42	14.85
Logger + Sex	Logger + Sex	177.22	15.65
Logger + Sex + Site	Logger + Sex + Site	177.27	15.70
Age + Logger + Sex + Site	Logger + Sex + Site	178.22	16.65
Age + Sex + Site	Sex + Site	11449.23	11287.66
Age + Logger + Site	Logger + Site	22756.73	22595.16
Age + Site	Site	24158.39	23996.82
Age + Logger + Sex	1	24161.13	23999.56
Age + Logger + Sex	Logger	24161.24	23999.67
Age + Logger + Sex	Logger + Sex	24164.53	24002.96
Age + Logger + Sex	Sex	24164.58	24003.01
Age + Sex	Sex	26980.05	26818.48

Age + Logger	Logger	29793.13	29631.56

Supplement 2

Details regarding geolocator deployment at four study regions in Russia. Given are the years when the study was conducted and the respective number of individuals.

Study region	Tagging years	Tagged	Recapture years	Returned	Recaptured	Retrieved
Amur	2016	18	2017/2018	8/1	4/0	3
Baikal	2018/2019	7/13	2019/2020/2021	3/4/1	1/4/1	6
Komi	2019	13	2020/2021	0/0	0/0	0
Volga	2019	3	2020	0	0	0

Supplement 3

Overview on persecution sites of Yellow-breasted Buntings since 2013.

Date	Site	N	Lat	Lon	Comments	Source
01/2024	Bago city, Bago, Myanmar	>2	17.34	96.51	Caged birds, captured locally for merit release	Thiri Dae We Aung, pers. obs.
01/2023	Bago city, Bago, Myanmar	>2	17.34	96.50	Caged birds, captured locally for merit release	Thiri Dae We Aung, pers. obs.
08-09/2021	Gaojia, Panjin, Lianoning, China	112	40.83	122.13	Live and dead birds confiscated at trapping site	http://rmfyb.chinacourt.org
05/09/2019	Linghai, Liaoning, China	12	41.11	121.14	Found in illegal mist-nets	Heim et al. 2021
03/09/2019	Huludao, Liaoning, China	52	40.75	120.83	Found in illegal mist-nets	Heim et al. 2021
11/12/2017	Mae Ai, Chiang Mai, Thailand	3	20.02	99.33	Found dead in mist-net	Heim et al. 2021
26/08/2017	Tangshan, Hebei, China	35	39.47	118.21	Collected from mist-nets	Heim et al. 2021

22/09/2013	Tianjin, China	100	39.08	117.20	Caged birds used to attract migrants	Kamp et al. 2015
18/09/2013	Nanpu, Tangshan, Hebei, China	1	39.04	118.27	Caged male to attract migrants	Kamp et al. 2015

Supplement 4

Estimated positions of nine individual Yellow-breasted Buntings tracked with light-level geolocators. For a legend regarding the seasonal colours, please refer to Figure 4 (red = breeding, beige = autumn stopover/moult, blue = non-breeding, gold = spring stopover). Whiskers indicate the standard deviation around the mean latitudes and longitudes.

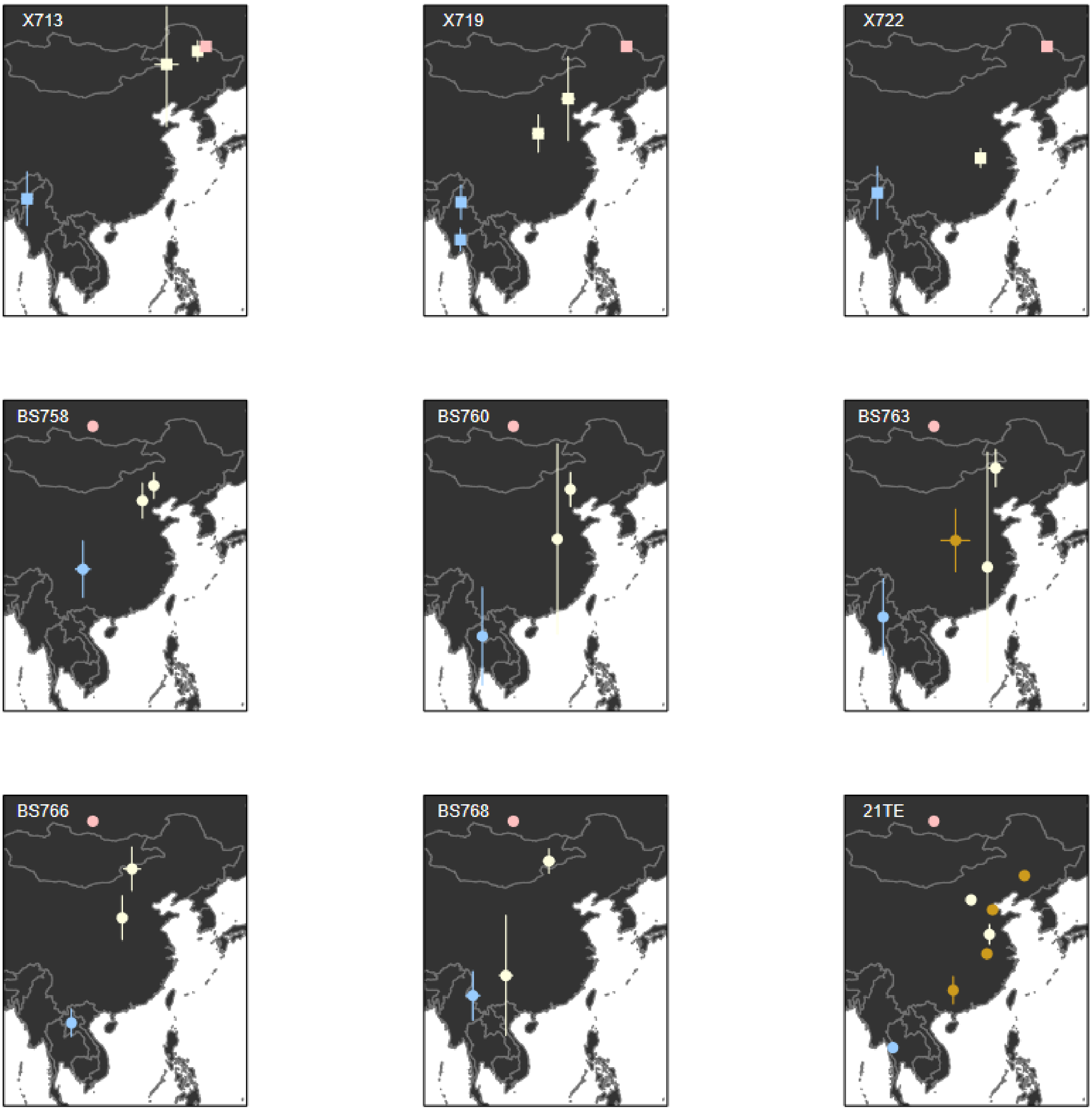
X713
X719
X722
BS758
BS760
BS763
BS766
BS768
21TE
